# Association between hepatitis A seropositivity and bone mineral density in adolescents and adults: a cross-sectional study using NHANES data

**DOI:** 10.1590/1516-3180.2023.0266.R1.08022024

**Published:** 2024-04-22

**Authors:** Zhuowen Yu, Gunchu Hu, Jiajie Wang, Zhihong Li

**Affiliations:** I Doctoral student, Physician. Department of Orthopedics, Second Xiangya Hospital of Central South University, Changsha, China; Hunan Key Laboratory of Tumor Models and Individualized Medicine, Second Xiangya Hospital of Central South University, Changsha, China.; II Doctoral student, Physician. Department of General Surgery, Second Xiangya Hospital of Central South University, Changsha, China.; III Master’s student, Physician. Department of Orthopedics, Second Xiangya Hospital of Central South University, Changsha, China; Hunan Key Laboratory of Tumor Models and Individualized Medicine, Second Xiangya Hospital of Central South University, Changsha, China.; IV PhD. Physician, Professor, Department of Orthopedics, Second Xiangya Hospital of Central South University, Changsha, China; Hunan Key Laboratory of Tumor Models and Individualized Medicine, Second Xiangya Hospital of Central South University, Changsha, China.

**Keywords:** Osteoporosis, Bone Density, Hepatitis A, Seroepidemiologic Studies, Nutrition Surveys, Adolescents, Adults, Multivariable regression

## Abstract

**BACKGROUND:**

Osteoporosis, characterized by decreased bone density and increased fracture risk, imposes significant physical, psychosocial, and financial burdens. Early detection and prevention are crucial for managing osteoporosis and reducing the risk of fractures.

**OBJECTIVES:**

To investigate the relationship between Hepatitis A seropositivity and bone mineral density (BMD) in adolescents and adults and to explore the potential link between Hepatitis A infection and osteoporosis risk.

**DESIGN AND SETTING:**

This cross-sectional study used data from the National Health and Nutrition Examination Survey (NHANES) from 2011 to 2018 to evaluate the association between hepatitis A seropositivity and BMD in 15,693 participants.

**METHODS:**

Multivariable regression analysis was used to calculate the mean BMD and standard error for adolescents and adults, followed by an independent z-test to determine whether there was a significant difference between the seropositive and seronegative groups.

**RESULTS:**

Hepatitis A seropositive adolescents and adults had lower BMD than their seronegative counterparts, with significant differences in lumber spine (mean difference = -0.03 g/cm2, P < 0.01 for both age groups) and pelvis BMDs (mean difference = -0.02 g/cm2, P < 0.01 for the adult age groups), after adjusting for various covariates.

**CONCLUSIONS:**

This study confirmed that both adolescent and adult individuals seropositive for Hepatitis A antibodies had reduced BMD among both adolescents and adults, especially in the adult group. This finding suggests a possible link between Hepatitis A infection and risk of osteoporosis.

## INTRODUCTION

Osteoporosis, a medical condition characterized by decreased bone density and increased fracture risk, often progresses silently until a fracture occurs.^1^ The condition primarily affects postmenopausal women and results from a loss of calcium and collagen in the bones.^2^ Notably, osteoporosis is not limited to any specific population or age group and can cause significant physical, psychosocial, and financial burdens across all populations and ages.^3-6^ Early detection and prevention are crucial for managing osteoporosis and reducing the risk of fracture.^2,7^

Bone mineral density (BMD) tests can aid in early detection by comparing an individual’s bone density to that of a healthy person of the same age and sex.^8^ The most commonly used BMD test is the central dual-energy x-ray absorptiometry (DXA) test, which measures the grams of calcium and other bone minerals packed into a segment of bone using X-rays. The spine, hip, and forearm are the most commonly tested bones.

Hepatitis A is an inflammation of the liver that can range from asymptomatic infection to severe illness.^9^ The Hepatitis A virus (HAV), a positive-strand RNA virus, is transmitted through ingestion of contaminated food and water or through direct contact with an infectious person.^9,10^ The incidence rate of reported Hepatitis A cases in the United States was one case per 100,000 population.^11^ Although almost everyone recovers fully from Hepatitis A with a lifelong immunity, a very small proportion of people infected with Hepatitis A could die from fulminant hepatitis.^12-14^ A safe and effective vaccine is available to prevent Hepatitis A.^9^

Previous studies have suggested that viral hepatitis may negatively affect bone metabolism and health. For example, chronic hepatitis B and C infections have been associated with lower BMD and a higher risk of osteoporosis in adults.^15-19^ Hepatitis A is a viral infection whose symptoms are clinically indistinguishable from other types of acute viral hepatitis.^20^ Therefore, it was hypothesized that Hepatitis A seropositivity may be related to a lower BMD and higher osteoporosis risk in adolescents and adults.

The National Health and Nutrition Examination Survey (NHANES), a program designed to assess the health and nutritional status of adults and children in the United States, combines interviews and physical examinations and covers a wide range of health and nutrition measurements. The survey examines a nationally representative sample of approximately 5,000 persons each year and collects data on various demographic, socioeconomic, dietary, and health-related factors. The survey results are used to determine the prevalence of major diseases and risk factors to assess nutritional status and their association with health promotion and disease prevention.^21^

This study aimed to investigate the relationship between Hepatitis A seropositivity and BMD in adolescents and adults using data from the NHANES. The potential link between Hepatitis A infection and osteoporosis risk was also explored, and the implications for bone health and prevention strategies are discussed.

## METHOD

### Design and setting

A cross-sectional design was used to examine the association between Hepatitis A seropositivity and BMD, which enabled the measurement of the prevalence of the outcome and exposure at a certain moment in time, and the identification of potential correlations.

### Data source and research participants

All patient information was based on data obtained from the NHANES. Four datasets (2011–2012, 2013–2014, 2015–2016, and 2017–2018) were used to combine the study population. The inclusion criteria required individuals to be tested for Hepatitis A antibodies (anti-HAV) and to undergo a DXA examination concurrently. Patients who were seropositive (+) for anti-HAV antibodies were compared to those who were seronegative (-).

### Subpopulation definitions

According to the World Health Organization (WHO), individuals between the ages of 10 and 19 years are classified as adolescents.^22^ Conversely, individuals > 19 years are considered adults. In the adult female population, those < 51 years are designated as premenopausal, while their counterparts are deemed postmenopausal, given that the average age of menopause in the United States is 51.^23^

### BMD testing

BMD testing for the full participant set (incorporated into the final evaluation) was performed using DXA examinations, which were performed by qualified and registered radiology technologists using Hologic Discovery model A densitometers (Hologic, Inc., Bedford, Massachusetts, USA) with the software version Apex 3.2. More specifics are furnished on the NHANES website.^24^ Lumbar spine and pelvis were chosen for evaluating the association as they have a higher risk of fracture compared to the skull bones, arms, legs, ribs, thorax and trunk bones.^25^

### Total serum anti-HAV assay

Serum specimens from the complete participant set (incorporated into the final evaluation) were processed, stored, and shipped to the Division of Viral Hepatitis, National Center for HIV/AIDS, Viral Hepatitis, STD, and TB Prevention, Centers for Disease Control and Prevention, Atlanta, GA for analysis. The Division uses the VITROS Immunodiagnostic Products Anti-HAV Total Reagent Pack and the VITROS Immunodiagnostic Products Anti-HAV Total Calibrator on the VITROS ECi/ECiQ or VITROS 3600 Immunodiagnostic System to detect IgG and IgM. Further details are provided in the NHANES Laboratory Procedures Manual.^26^

### Covariates

Covariates were added to improve the accuracy of this study; these are variables that are not of primary interest but can influence the outcome of a study.^27,28^ In the present study, several covariates were considered for both adult and adolescent populations. For adults, age, race, education level, income level, body mass index (BMI), smoking status, alcohol consumption, diabetes, physical activity level, and serum 25-hydroxyvitamin D [25(OH)D] levels were included as covariates. For adolescents, the same covariates were considered with the exception of smoking status and alcohol consumption. Detailed data on the covariates are listed in Supplemental Table 1.

### Statistical evaluation

All evaluations were based on the participants’ complete data. Individuals with missing covariate data were excluded from the final evaluation. The NHANES sample weights assigned by the Centers for Disease Control and Prevention (USA) based on the sample design for each survey year were used. Therefore, as some of the variables included in this study were captured at the mobile examination center (MEC), we used the MEC exam weight (WTMEC2YR) for evaluation.

Furthermore, the sample weight used in the final evaluation was equal to one-fourth the value of “WTMEC2YR” because we combined four NHANES survey cycles. Baseline characteristics were indicated by the weighted mean and standard error (SE) (continuous variables) and weighted proportion (categorical variables). Furthermore, since the four NHANES survey cycles were combined, the sample weight used in the final evaluation was equal to one-fourth the value of “WTMEC2YR”. The baseline characteristics are presented as weighted proportions (categorical variables) and weighted means and SE (continuous variables). The weights used for these evaluations were chosen according to the guidelines delineated in the NHANES database.^29^

Multivariable regression analysis for BMD was implemented with adjustment for age, race, and BMI in Model 1, and adjusted for age, race, education level, income level, BMI, smoking status, alcohol consumption, diabetes, physical activity level, and serum 25(OH)D levels in Model 2.

Kolmogorov–Smirnov tests were used to determine whether the BMD variables conformed to the normal distribution, and logarithmic transformation was utilized for those that did not conform to the normal distribution.

Independent z-tests were used to assess the statistical significance of differences in BMD between the seropositive and seronegative groups for Hepatitis A antibodies.

All evaluations were performed using R software (version 4.2.3; https://www.R-project.org). Statistical significance was set at P < 0.05.

## RESULTS

### Segmentation and baseline characteristics

**Figure 1:**
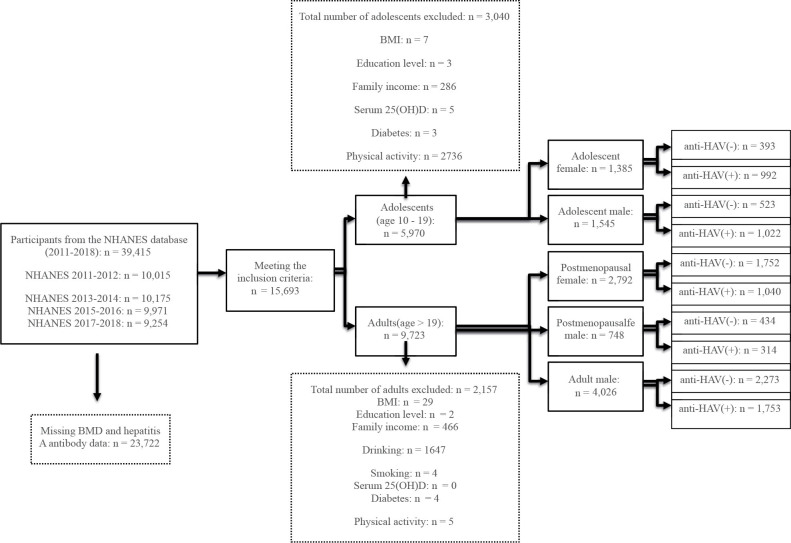
Flow chart of the study design.

A flowchart of the segmentation process is shown in Figure 1. Data from 39,415 participants were obtained from the NHANES over four cycles: 2011–2012 (n = 10,015), 2013–2014 (n = 10,175), 2015–2016 (n = 9,971), and 2017–2018 (n = 9,254).

First, following the elimination of 23,722 participants from the original 39,415 owing to missing data on BMD or Hepatitis A antibodies, 15,693 participants meeting the inclusion criteria were included for subsequent analysis. Each participant’s Hepatitis A antibody status and BMD were evaluated concurrently within these respective cycles to ensure temporal alignment of the measurements.

Second, the participants were stratified into adolescents (n = 5,970) and adults (n = 9,723) based on age. The covariates for adults included age, race, education level, income level, BMI, smoking status, alcohol consumption, diabetes, physical activity level, and serum 25 (OH) D levels. For adolescents, the covariates were the same except for smoking and alcohol use, which are illegal for minors. After excluding participants with missing covariates, 2,930 adolescents and 7,566 adults were included for further analysis.

Third, stratification of the adolescent and adult participants by sex yielded five groups: adolescent females (n = 1,385) and males (n = 1,545), premenopausal (n = 2,792) and postmenopausal (n = 748) adult females, and adult males (n = 4,026). Each of these five groups was then dichotomized as having either seronegative or seropositive Hepatitis A antibody status, providing 10 categories for subsequent analysis.

The baseline characteristics of the groups are presented in Table 1. In both adolescents and adults, the weighted mean age of the seronegative individuals was higher than that of the seropositive individuals. Specifically, the seronegative adolescents had a weighted mean age of 15.83 ± 0.10 years compared with 15.43 ± 0.07 years of the seropositive adolescents. Similarly, the seronegative adults had a weighted mean age of 39.91 ± 0.23 years compared with 37.78 ± 0.29 years of the seropositive adults. Regardless of age, the weighted proportion of non-Hispanic whites in the seropositive population was lower than that in the seronegative population (Supplemental Figure S1). Furthermore, the seronegative individuals were found to have higher levels of weighted mean serum 25(OH)D levels than the seropositive individuals in both age groups; detailed information on other variables is available in Table 1 **with P values in** Supplemental Table S2.

**Table 1:** aseline characteristics of participants incorporated into the final evaluation

		Adolescents		Adults	
		anti-HAV(-) (n = 916)	anti-HAV(+) (n = 2014)	anti-HAV(-) (n = 4459)	anti-HAV(+) (n = 3107)
Sex, n (%)	Female	414 (45.15)	971(48.22)	**2180 (48.90)**	**1345 (43.28)**
	Male	502 (54.85)	1043 (51.78)	**2279 (51.10)**	**1762 (56.72)**
Age [year], mean (SE)		**15.83 (0.10)**	**15.43 (0.07)**	**39.91 (0.23)**	**37.78 (0.29)**
Race, n (%)	Non-Hispanic white	**622 (67.93)**	**993 (49.31)**	**3323 (74.52)**	**1381 (44.43)**
	Mexican American	**51 (5.53)**	**410 (20.37)**	**191 (4.28)**	**668 (21.51)**
	Other Hispanic	**42 (4.64)**	**180 (8.92)**	**176 (3.94)**	**394 (12.67)**
	Non-Hispanic black	**136 (14.83)**	**235 (11.66)**	**478 (10.72)**	**282 (9.08)**
	Other races	**65 (7.07)**	**196 (9.74)**	**291 (6.54)**	**382 (12.31)**
Annual family income, n (%)	Under $20,000	153 (16.72)	358 (17.77)	**623 (13.98)**	**546 (17.57)**
	$20,000 and over	763 (83.28)	1656 (82.23)	**3836 (86.02)**	**2561 (82.43)**
Education level, n (%)	Lower than 5th grade	**30 (3.26)**	**86 (4.25)**	-	-
	5th grade to 8th grade	**423 (46.21)**	**1056 (52.44)**	-	-
	Higher than 8th grade	**463 (50.53)**	**872 (43.31)**	-	-
	Below high school	-	-	**406 (9.11)**	**530 (17.05)**
	High school or equivalent	-	-	**981 (22.00)**	**651 (20.96)**
	Above high school	-	-	**3072 (68.89)**	**1926 (61.99)**
BMI [kg/m2] , mean (SE)		24.52 (0.28)	23.99 (0.16)	**28.93 (0.12)**	**28.06 (0.14)**
Diabetes, n (%)	Yes	4 (0.38)	9 (0.44)	250 (5.60)	162 (5.21)
	No	907 (99.16)	1992 (98.93)	4136 (92.76)	2899 (93.29)
	Borderline	5 (0.46)	13 (0.63)	73 (1.64)	46 (1.50)
Serum 25(OH)D [nmol/L], mean (SE)		**65.16 (0.98)**	**62.54 (0.77)**	**68.51 (0.50)**	**64.10 (0.70)**
High-risk drinking, n (%)	Yes	-	-	**716 (16.05)**	**412 (13.25)**
	No	-	-	**3743 (83.95)**	**2695 (86.75)**
Smokers, n (%)	Yes	-	-	**2113 (47.38)**	**1219 (39.23)**
	No	-	-	**2346 (52.62)**	**1888 (60.77)**
AST [U/L], mean (SE)		23.91 (0.65)	23.87 (0.38)	25.08 (0.32)	26.04 (0.45)
ALT [U/L], mean (SE)		19.84 (0.74)	19.41 (0.37)	**25.46 (0.34)**	**27.83 (0.50)**

% = weighted proportion. 25(OH)D = 25-hydroxyvitamin D; AST = aspartate transaminase; ALT = alanine transaminase; BMI = body mass index; SE = standard error; HAV = hepatitis A virus; High-risk drinking = men who consumed more than five drinks every day and women who consumed more than four drinks every day; smokers = smoking at least 100 cigarettes in life.

Bold indicates statistical difference.

### Adolescents’ association between anti-HAV and BMD

Lumbar spine and pelvic BMDs of all subpopulations were tested using the Kolmogorov–Smirnov test to determine whether they conformed to the normal distribution. Those who did not conform to the normal distribution at first conformed to the normal distribution after logarithmic transformation.

Multivariable regression analysis was used to calculate the mean BMD and SE for adolescents (Table 2). Two models were used: Model 1, adjusted for age, race, and BMI; and Model 2, adjusted for age, race, BMI, education level, annual family income, diabetes, physical activity level, and serum 25(OH)D levels. Whether BMD was more likely to be lower in the anti-HAV (+) participants than in the anti-HAV (-) participants was determined using independent z-tests with a statistical significance threshold of P < 0.05. The results showed that lumbar spine BMD was significantly lower in the anti-HAV (+) adolescent males than in the anti-HAV (-) adolescent males in both models (Model 1: mean difference = -0.018 g/cm2, P = 0.002; Model 2: mean difference = -0.021 g/cm2, P < 0.001). No other significant differences were observed between groups.

**Table 2:** Association between anti-hepatitis A virus and bone mineral density in adolescents

	Model 1			Model 2		
	anti-HAV (-)	anti-HAV (+)	P	anti-HAV (-)	anti-HAV (+)	P
Lumbar Spine						
Female	0.999 ± 0.003	0.995 ± 0.002	0.343	1.002 ± 0.004	0.996 ± 0.002	0.142
Male	0.955 ± 0.005	0.937 ± 0.003	**0.002**	0.958 ± 0.005	0.937 ± 0.003	**<0.001**
Pelvis						
Female	1.182 ± 0.004	1.190 ± 0.003	0.134	1.187 ± 0.004	1.191 ± 0.003	0.476
Male	1.183 ± 0.006	1.174 ± 0.004	0.206	1.188 ± 0.006	1.175 ± 0.004	0.071

Model 1: Adjusted for age, race (non-Hispanic white, Mexican American, other Hispanic, non-Hispanic black, and other races), and body mass index.

Model 2: Adjusted for age, race (non-Hispanic white; Mexican American; other Hispanic; non-Hispanic black; other races), body mass index, education level (lower than 5^th^ grade; 5^th^ grade to 8^th^ grade; higher than 8^th^ grade), annual family income (under $20,000; $20,000 and over), diabetes (yes, no, borderline), physical activity level (vigorous work activity, moderate work activity, walking or cycling, vigorous recreational activities, and moderate recreational activities), and serum 25-hydroxyvitamin D levels.

HAV = hepatitis A virus.

All bone mineral density scores are in g/cm^2^.

Bold indicates statistical difference.

### Adults’ association between anti-HAV and BMD

**Figure 2:**
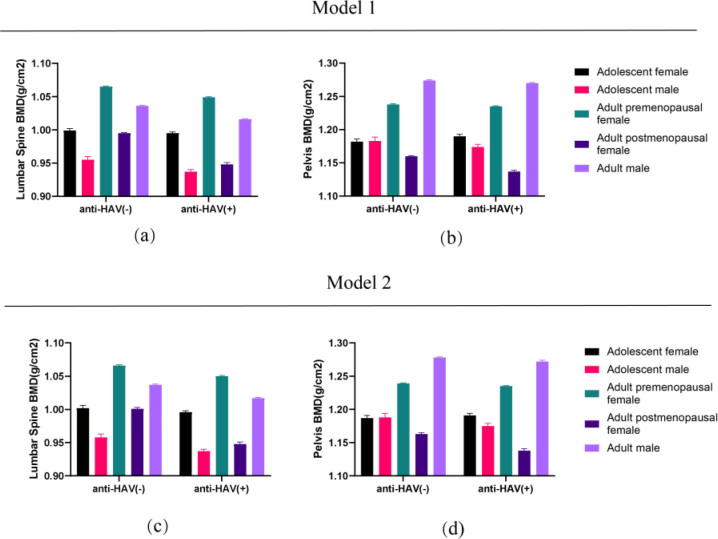
Comparative analysis of lumbar Spine and pelvis bone mineral density in two models across 10 subpopulations. **(a)** Lumbar spine bone mineral density distribution in model 1; **(b)** Pelvis bone mineral density distribution in model 1; **(c)** Lumbar spine bone mineral density distribution in model 2; **(d)** Pelvis bone mineral density distribution in model 2. The height of each histogram represents the mean value and the bars represent the standard error.

Adults, divided into premenopausal and postmenopausal females and males were examined using multivariable regression analysis to calculate their mean BMD and SE (Table 3). Two models were used: Model 1, adjusted for age, race, and BMI; and Model 2, adjusted for age, race, BMI, education level, annual family income, diabetes, physical activity level, high-risk drinking, smoking, and serum 25(OH)D levels. Independent z-tests were used to determine whether BMD was more likely to be lower in anti-HAV (+) participants than in anti-HAV (-) participants, with a statistical significance threshold of P < 0.05. The results showed that the lumbar spine and pelvic BMDs were significantly lower in the anti-HAV (+) participants than in the anti-HAV (-) participants in all groups (premenopausal females, postmenopausal females, and males) in both models (all P < 0.05). Lumbar spine and pelvis BMDs of Models 1 and 2 for all subpopulations of adolescents and adults are illustrated in Figure 2.

**Table 3:** Association between anti-hepatitis A virus and bone mineral density in adults

	Model 1			Model 2		
	anti-HAV(-)	anti-HAV(+)	P	anti-HAV(-)	anti-HAV(+)	P
Lumbar Spine						
Pre	1.065 ± 0.001	1.049 ± 0.001	**< 0.001**	1.066 ± 0.001	1.050 ± 0.001	**< 0.001**
Post	0.995 ± 0.001	0.948 ± 0.003	**< 0.001**	1.001 ± 0.002	0.948 ± 0.003	**< 0.001**
Male	1.036 ± 0.001	1.016 ± 0.001	**< 0.001**	1.037 ± 0.001	1.017 ± 0.001	**< 0.001**
Pelvis						
Pre	1.238 ± 0.001	1.235 ± 0.001	**0.019**	1.239 ± 0.001	1.235 ± 0.001	**0.028**
Post	1.160 ± 0.001	1.137 ± 0.002	**< 0.001**	1.163 ± 0.002	1.138 ± 0.003	**<0.001**
Male	1.274 ± 0.001	1.270 ± 0.001	**0.005**	1.278 ± 0.001	1.272 ± 0.002	**<0.001**

Model 1: Adjusted for age, race (non-Hispanic white, Mexican American, other Hispanic, non-Hispanic black, and other races), and body mass index.

Model 2: Adjusted for age, race (non-Hispanic white; Mexican American; other Hispanic; non-Hispanic black; other races), body mass index, education level (below high school, high school, or equivalent; above high school), annual family income (under $20,000; $20,000 and over), diabetes (yes, no, borderline), physical activity level (vigorous work activity; moderate work activity; walk or bicycle; vigorous recreational activities; moderate recreational activities), high-risk drinking, smoking, and serum 25-hydroxyvitamin D levels.

HAV = hepatitis A virus.

All bone mineral density scores are in g/cm^2^

Bold indicates statistical difference.

Pre premenopausal female, Post postmenopausal female.

## DISCUSSION

Examination of the relationship between Hepatitis A seropositivity and BMD revealed that both adolescents and adults who tested positive for Hepatitis A antibodies had lower BMDs than their seronegative counterparts. This association persisted even after controlling for several covariates that differed between the two groups, suggesting an increased risk of osteoporosis in patients with Hepatitis A seropositivity. Bone mineral density is a measure of the amount of minerals such as calcium and phosphorus in bones, which are essential for maintaining bone strength and preventing fractures. Hepatitis A can cause an increase of liver enzymes to above normal levels in the bloodstream, similar to other types of acute viral hepatitis, indicating liver dysfunction and a risk of liver damage.^30^ Therefore, it may have a negative effect on nutrient metabolism absorption. Besides, the cytokines including interleukin-10, interleukin-1α, interleukin-6, interleukin-8, interleukin-13, tumor necrosis factor-alpha, and transforming growth factor-beta, are released in a Hepatitis A infection.^31-33^ These cytokines may increase the risk of reduction of BMD by stimulating the receptor activator of nuclear factor κ-β ligand-receptor activator of nuclear factor κ-β-osteoprotegerin (RANKL-RANK-OPG) system, which regulates the balance between bone formation and resorption.^34^ Multivariable regression is a method used to construct models that investigate the statistical relationship between a response variable (Y) and explanatory variables (Xi), with multivariable linear regression used when Y is continuous and approximately normal.^35^ In this study, Y was the BMD of the lumbar spine or pelvis, which are continuous variables, and the covariates were considered as Xi. Therefore, it was reasonable to use multivariable linear regression to construct the two models, in which the means and SEs of the lumbar spine and pelvic BMDs were calculated after adjusting for covariates. The association in adolescents was less significant than that in adults, which may be attributable to the bone growth features of adolescents. Although adolescents and adults experience different changes in BMD, BMD is important in both age groups. During adolescence, bone growth occurs rapidly, leading to an increase in overall BMD. This process slows down considerably after the age of approximately 20 years, when most people reach peak bone mass (PBM).^36^ In contrast, adults face various factors that can negatively affect their BMD, including aging, hormonal changes (e.g., menopause), certain medications, or chronic diseases like osteoporosis.^37^ The results illustrated in Figure 2 demonstrate that adolescents have lower lumbar spine and pelvic BMDs than adults (premenopausal females included), and postmenopausal women have lower BMD than premenopausal women in the same model and anti-HAV subgroup. These results confirm the robustness of the multivariable linear regression analysis. As people age, their bones tend to lose density, making them more prone to breakage, even with minimal trauma. Poor bone mass acquisition during growth, leading to lower than optimal levels of PBM, increases the risk of developing osteoporosis or other related conditions.^36,38,39^ Moreover, osteoporosis is not only common in white postmenopausal women as it occurs in other populations of all ages, with significant physiological, mental, and economic consequences.^3-6^ Therefore, it is important for individuals of all ages to prevent long-term complications associated with low BMD levels, regardless of whether they are adolescents or adults.

In this study, a link between Hepatitis A and bone loss was discovered for the first time; however, similar findings have been reported for other forms of hepatitis, with several studies showing an association between osteoporosis and Hepatitis B. Patients with a Hepatitis B infection tend to experience bone loss and may even develop osteoporosis or bone fractures. Zhang et al. found that patients with Hepatitis B cirrhosis had lower BMD and higher prevalence of osteoporosis than healthy controls.^18^ Dessordi et al. reported that patients with a Hepatitis B infection had lower BMD and higher serum levels of osteoclast markers than controls, suggesting that chronic viral infection may enhance bone resorption independently of antiretroviral therapy.^16^ Oh et al. reported that patients with chronic Hepatitis B infection in South Korea had increased risks of osteoporosis compared to the general population, as evidenced by a 9% higher fracture rate and an upward trend of osteoporotic fractures from 2007 to 2016.^17^ Baeg et al. reported that male patients with Hepatitis B virus infection had lower BMD in the femur and lumbar spine than those without the infection.^15^ Wei et al. reported that antiviral therapy for chronic Hepatitis B with tenofovir disoproxil fumarate or entecavir did not increase the risk of osteopenia or osteoporosis in Asian patients during a median follow-up of 4–5 years.^40^ Research on Hepatitis C and other chronic active hepatitis has also shown a loss of bone. Wijarnpreecha et al., in a meta-analysis of four studies, reported that Hepatitis C virus infection was associated with an increased risk of osteoporosis.^19^ Clements and Rhodes reported a case of a 41-year-old female patient with chronic active hepatitis who had a high rate of bone loss, in the context of increased incidence of osteoporosis in patients with chronic liver disease.^41^ Hepatic osteodystrophy (HOD), a condition of bone loss in patients with chronic liver diseases, which leads to an increased risk of osteoporosis and osteoporotic fractures, has been proposed as a theory to explain this phenomenon. The pathogenesis of HOD is complex and multifactorial, and involves hormonal, inflammatory, nutritional, and genetic factors.^34, 41-44^

Hepatitis A is an acute disease that does not result in a chronic infection,^10^ unlike HOD, which occurs only in patients with chronic liver disease. The anti-HAV assay used in this study measures total anti-HAV (IgG or IgM) in human serum or plasma, indicating past or present infection with HAV or vaccination against HAV.^26^ Interestingly, the incidence rate of reported Hepatitis A cases in the United States (one case per 100,000 population) was significantly lower than the seropositive rate of anti-HAV in the study population (68.74% in adolescents and 41.06% in adults). This suggests that the high seropositivity rate of anti-HAV may not be due to infection but rather vaccination. However, the association between vaccination and bone loss has not yet been reported.

The present study was limited by the use of a total serum anti-HAV assay that detected total anti-HAV antibodies (including IgG or IgM). This assay does not allow for the differentiation of IgG and IgM, which can affect the BMD. Additionally, the study was cross-sectional, which means that it was not possible to establish the temporality between HAV infection and reduced BMD. Finally, this study did not assess whether a reduction in BMD leads to clinically relevant diseases. These limitations should be considered when interpreting the results of this study.

## CONCLUSIONS

This study confirmed that Hepatitis A seropositivity was associated with reduced BMD in both adolescents and adults, suggesting that it is a potential risk factor for osteoporosis. Individuals seropositive for Hepatitis A should be aware of this risk and take preventive measures. Further research is needed to verify the causal effects of Hepatitis A antibodies on bone tissue and elucidate the underlying mechanisms. These findings may have implications for public health and inform the development of targeted interventions to prevent osteoporosis.

## SUPPLEMENTARY MATERIAL:

Supplementary material form this article can be seen in the link: https://github.com/FearlessYu/SPMJ-2023-0266.R1.Supplementary-material.git
